# Succus Taraxaci Paratus

**Published:** 1853-09

**Authors:** 


*Succus Taraxaci Paratus.*—The preparations of Taraxacum, or *dandelion* root, have within a few years past attracted considerable attention from the medical profession, and have been an object of experiment from skilful pharmaceutists both in England and the United States. The hurtful effect exercised by heat in evaporating solutions of the soluble matter of dandelion, has long been noted, and many recollect the molasses-like extract which formerly was too commonly seen, unmarked by any distinct, sensible property of the recent root. The application of spontaneous and vacuum operation in making the solid extract, demonstrated this beyond cavil, and in the last U. S. Pharmacopœia directions were given for this precaution, as well as in regard to the period of collecting the root. The *Liquor Taraxaci* (fluid extract) described by Mr. Redwood in Gray's Supplement, is prepared by macerating four pounds of the recently dried root in sufficient cold water for 24 hours, expressing, and evaporating to 36 fluid ounces, to which liquid 12 fluid ounces of alcohol is added; hence each fluid ounce of the preparation represents a troy ounce of the *dried* root. The dried root of the dandelion is more easily extracted than when it is recent, owing to the glutinous character of the juices and the difficulty of effectually disintegrating the tissue of the root, so as to completely reach the contained matter by menstrua; yet it is more than probable that the evaporation of the aqueous solution to the proper degree of concentration, not to speak of the effect of the process of drying the root in the first place, injnres the product. This is partially avoided by a process published in the 20th volume, page 86 of the Journal of Pharmacy, the *recent root* being employed, and the resulting preparation representing about twice its weight of the fresh root. In the preparation about to be described, the virtues of the root, be they what they may, are preserved unimpaired, as no heat is employed in the process; the natural juice of the plant being merely admixed with alcohol to preserve it from change. Take of fresh Dandelion Roots (collected in September or October) twenty pounds, (*av.*) Alcohol, 835, sp. gr. four pints. Slice the roots transversely in short sections, and by means of a mill or mortar and pestle reduce them to a pulpy mass; then add the alcohol and mix them thoroughly.

The mixture thus far prepared at the season when the root is proper for collection, may be set aside in suitable vessels; stoneware jars are appropriate; and extracted as the preparation is needed through the other seasons. After having stood a week, or until a convenient time, the pulpy mass is subjected to powerful pressure, until as much as possible of the fluid is removed. This is then filtered and bottled for use. It is necessary that sufficient time should elapse after the pulp is set aside for the alcohol to penetrate the fibrous particles and commingle with the natural juices, as well as for the woody structure of the root to loose its elasticity, that it may yield the juice more completely on pressure. When the pulp has stood six months in this manner it yields the juice with great readiness, and is possessed of the sensible propertes of the dandelion in a marked degree. When eight pounds, avoirdupois, of the root is thus treated, after standing several months, the practical result is about six pints of fluid, with an ordinary screw-press. This yield will vary in amount with the condition of the root when collected, and the length of time it is exposed afterwards, as well as the power of the press used. Should the alcohol in this preparation be contraindicated, it might be partially removed by exposure in a water bath until the juice was reduced to five-sixths of its bulk, and then for every pint of the residue eight ounces Troy of sugar may be added and dissolved in it.—*American Journal of Pharmacy.*

